# Exploring secretory proteome and cytokine kinetic of human peripheral blood mononuclear cells exposed to methicillin-resistant *Staphylococcus aureus* biofilms and planktonic bacteria

**DOI:** 10.3389/fimmu.2024.1334616

**Published:** 2024-03-20

**Authors:** Reza Gheitasi, Daniela Röll, Mario M. Müller, Mohadeseh Naseri, Rainer König, Hortense Slevogt, Mathias W. Pletz, Oliwia Makarewicz

**Affiliations:** ^1^ Institute of Infectious Diseases and Infection Control, Jena University Hospital/Friedrich Schiller University, Jena, Germany; ^2^ Septomics Research Center, Jena University Hospital, Jena, Germany; ^3^ Integrated Research and Treatment Center - Center for Sepsis Control and Care (CSCC), Jena University Hospital, Jena, Germany; ^4^ Respiratory Infection Dynamics, Helmholtz Centre for Infection Research-HZI Braunschweig, Braunschweig, Germany; ^5^ Department of Respiratory Medicine and Infectious Diseases, Hannover Medical School, German Center for Lung Research (DZL), Biomedical Research in Endstage & Obstructive Lung Disease (BREATH), Hannover, Germany; ^6^ CAPNETZ STIFTUNG, Hannover, Germany

**Keywords:** biomarkers, machine learning, chronic infections, proteomics, artificial neural networks, cytokine profiling, IL-18

## Abstract

*Staphylococcus aureus* is a highly successful pathogen infecting various body parts and forming biofilms on natural and artificial surfaces resulting in difficult-to-treat and chronic infections. We investigated the secreted cytokines and proteomes of isolated peripheral blood mononuclear cells (PBMCs) from healthy volunteers exposed to methicillin-resistant *S. aureus* (MRSA) biofilms or planktonic bacteria. Additionally, the cytokine profiles in sera from patients with community-acquired pneumonia (CAP) caused by *S. aureus* were investigated. The aim was to gain insights into the immune response involved and differentiate between the planktonic and sessile MRSA forms. We identified 321 and 298 targets that were significantly differently expressed in PBMCs when exposed to planktonic or biofilm-embedded bacteria, respectively. PBMCs exposed to planktonic MRSA cells secreted increased levels of TNF-α, while IL-18 was elevated when exposed to the biofilm. The machine-learning analyses of the cytokine profiles obtained for the *in vitro* PBMCs and CAP sera distinguished between the two types of bacteria forms based on cytokines IL-18, IL12, and IL-17, and with a lower importance IL-6. Particularly, IL-18 which has not been correlated with *S. aureus* biofilms so far might represent a suitable marker for monitoring chronification during MRSA infection to individualize the therapy, but this hypothesis must be proved in clinical trials.

## Highlights

- Analysis of peripheral blood mononuclear cells (PBMCs) exposed to methicillin-resistant *Staphylococcus aureus* (MRSA) biofilms and planktonic bacteria revealed distinct cytokine profiles and secretory proteome patterns.
- Elevated level of TNF-α was observed in PBMCs exposed to planktonic MRSA cells, while IL-18 was increased in response to biofilm exposure.
- Machine-learning analyses of cytokine profiles distinguished between planktonic and biofilm-embedded MRSA mainly based on IL-18, IL-12, and IL-17.
- IL-18 was so far not correlated to biofilm-associated infections with MRSA.

## Introduction

*Staphylococcus aureus* is a Gram-positive bacterium that can cause a wide range of infections in humans, ranging from minor infections to life-threatening diseases such as infective endocarditis (IE), bone and joint infections, and sepsis (1)*. S. aureus* infections are a major public health concern, with methicillin-resistant *S. aureus* (MRSA) responsible for many hospital-associated infections each year (2). *S. aureus* is known to form complex communities known as biofilms that adhere to surfaces and are embedded in a matrix of extracellular polymeric substances (EPS) composed of poly-N-acetylglucosamine (PNAG), proteins, and extracellular DNA (eDNA) (3). These offer protection against immune reactions and high tolerance to antibiotic treatments, which often make antimicrobial therapies ineffective (4, 5). Biofilms arise primarily on medical devices such as catheters and implants and are difficult to eliminate, which is usually only possible through surgery and the removal of the infected part. In addition, bacteria from biofilms can spread through the bloodstream and colonize other organs or implants leading to persistent infections (6). In recent years, it was shown that the host immune response to biofilms differs from that of planktonic cells and that biofilms are capable of reducing immune recognition (7, 8).

The immune response against bacteria in chronic infections is complex and involves both the innate and adaptive immune systems (9, 10). The innate immune response is the first line of defense and is activated by the pattern recognition receptor (PRR) pathways that detect general but bacterial-specific markers such as lipoproteins, peptidoglycans, and their breakdown products, or unmethylated CpG motifs in the DNA by different specialized Toll-like receptor (TLR) (11–13). This leads to the activation of phagocytic cells, such as macrophages and neutrophils, and the production of proinflammatory cytokines, such as IL-1, IL-6, IL-12p70, IL-18, and TNF, by host innate immune cells, including monocytes. The adaptive immune response is activated later during the course of infection. It depends on the presentation of bacterial antigens by antigen-presenting cells and is influenced by the cytokine milieu generated by the innate response (14). T-cell activation is an important component of the adaptive immune response, which targets specific bacterial antigens and can be recalled during subsequent infections to provide ‘memory’ against that particular pathogen. In the context of *staphylococcal* infection, T helper cells (Th cells) play a critical role in coordinating the adaptive immune response and producing proinflammatory cytokines (15). One such cytokine is IL-16, which acts as a chemoattractant for T cells and helps regulate their growth and responsiveness to regulatory cytokines. IL-16 levels are higher in *S. aureus*-infected mice and human monocytic cells compared to Gram-negative bacteria (16). *S. aureus* surface virulence factor protein A (SpA) activates TNFR1 signaling and IL-16 processing in various cell types. However, little is known about the role of IL-16 in the pathogenesis of bacterial infection. Understanding the immune response to biofilms is critical for developing effective diagnostics and treatments for biofilm-associated infections, as bacteria in biofilms differ in their resistance to antibiotics and host immune response.

## Results

### Formation of *S. aureus* biofilms in RPMI medium

**Figure 1:**
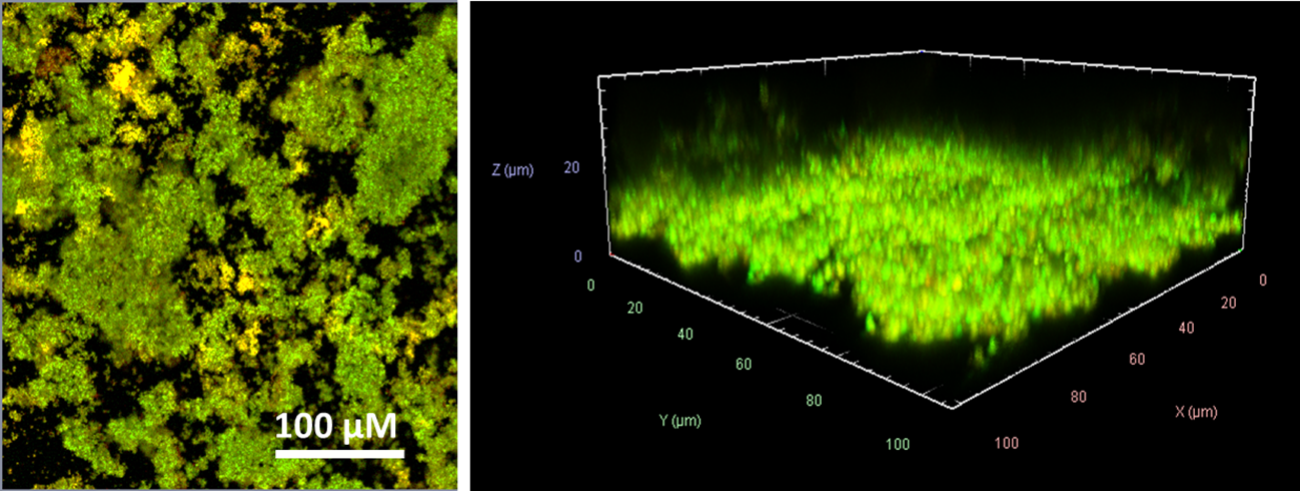
CLSM visualization of the *S. aureus* ATCC 43300 biofilms formed in RPMI medium. SYTO9^®^ (green) refers to viable cells, and propidium iodide (red) refers to dead cells. Left side: one layer each within biofilm at 100x magnification; right side: 3D visualization of the recorded Z-stack at 400x magnification.

For all *in vitro* experiments, the laboratory standard *S. aureus* ATCC 43300 with methicillin-resistant phenotype (MRSA) was used. To prove that *S. aureus* ATCC 43300 forms biofilms in a medium compatible with eukaryotic cells, the bacteria were incubated in RPMI 1640 medium and supplemented with 10% heat-inactivated fetal bovine serum (FBS) and 2 µM L-glutamine (enriched RPMI) at 37°C in humidified conditions for 24 h. After fluorescent live/dead-staining, the vitality and structure of the biofilms were examined using confocal laser scanning microscopy (CLSM) (**Figure 1**). The biofilms showed a dense SYTO9^®^ (green) stained biomass that formed a diffuse layer of approximately 10-20 µm in size. SYTO9^®^ is a membrane-permeable cationic cyanine dye that intercalates with nucleic acids staining almost all cells. Isolated propidium iodide (red) stained cells indicated dead or damaged cells. Propidium iodide can only penetrate the cell when the cytoplasm membrane becomes permeable, e.g., during membrane damage or disruption. Due to the presence of bacteria in various growth phases within the biofilm, including some that may have already undergone cell death after 24 h even when grown in common bacteria media, such as Luria-Bertani (LB) or Mueller-Hinton (MH) broth (17), it is expected that a portion of the cells become stained red. As most of the biomass is green stained and thus viable, we were convinced that we could use the enriched RPMI for biofilm formation in our experiments.

### Time-dependent changes in cytokine profiles of challenged PBMCs

Isolated PBMCs were applied on 24 h old MRSA biofilms or mixed with planktonic MRSA suspensions (both conditions, in a ratio of 1/20) and co-cultured for a further 24 h. We assessed the time-dependent cytokine kinetics of the PBMCs at 2, 4, 8, and 24 hours of 12 cytokines (details see method section) by a bead-based multiplex assay and compared the cytokine pattern to that of unchallenged PBMCs cultured under the same conditions (control group).

**Figure 2:**
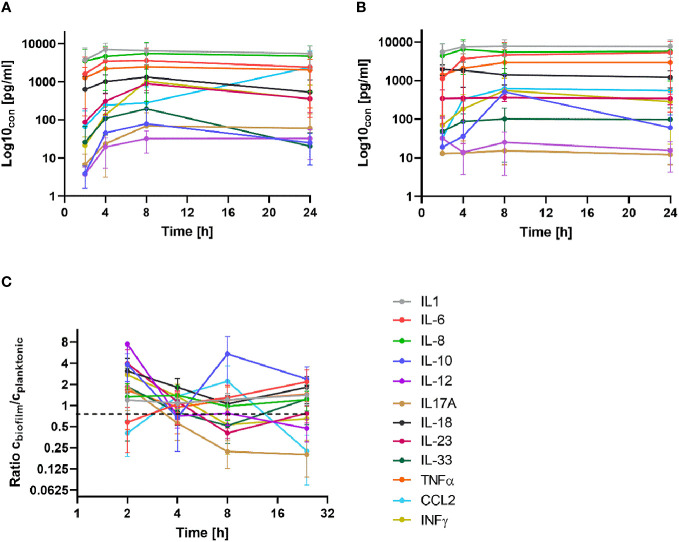
Time-dependent secretion of cytokines in BPMCs exposed to planktonic MRSA **(A)** and its biofilms **(B)**. **(C)** The ratio of the cytokine concentrations (biofilm/planktonic) plotted against time (as log2) for better visualization of the differences. The bars in **(A, B)** indicate the standard deviation (SD); in **(C)**, the bars represent the standard error of the mean (SEM). The legend is given in **(C)** for all diagrams. A detailed visualization of the individual cytokines in comparison to the control group is available in **Supplementary Figure S1**.

All tested cytokines of the PBMCs exposed to planktonic MRSA visibly increased in time time-dependent manner until 8 h of exposure (**Figure 2A**). After 24 h, the levels of some of the cytokines decreased compared to the 8 h levels but still were above the levels at the beginning of exposure (2 h). The cytokine levels of the PBMCs challenged with MRSA biofilms (**Figure 2B**) showed more variance: INFγ and IL-6 levels were not affected, IL-12 and IL-18 levels dropped down within 24 h, and IL-10 and CCL2 concentrations increased sharply up to 8 h but IL-10 decreased somewhat after 24 h, while other cytokines increased until 8 h and stayed stable for 24 h. To better visualize the differences in the cytokine tendencies over time, we plotted their ratios (as c_biofilm_/c_planktonic_) against a log2 representation of the time (**Figure 2C**). This demonstrated clearly that most cytokines were secreted at the same or lower levels when the PBMCs were stressed with biofilms compared to planktonic MRSA. Only IL-10 was visibly increased due to the biofilms. CCL2 concentration was only higher after 8 hours in the presence of biofilms but strongly increased when planktonic MRSA was present, inverting the ratio after 24 h. However, due to the high variability of the determined cytokine concentrations within the time-dependent repetitions, no significance could be determined in the experimental data, which complicates the interpretation.

### Differential cytokine responses to MRSA biofilms and planktonic bacteria

**Figure 3:**
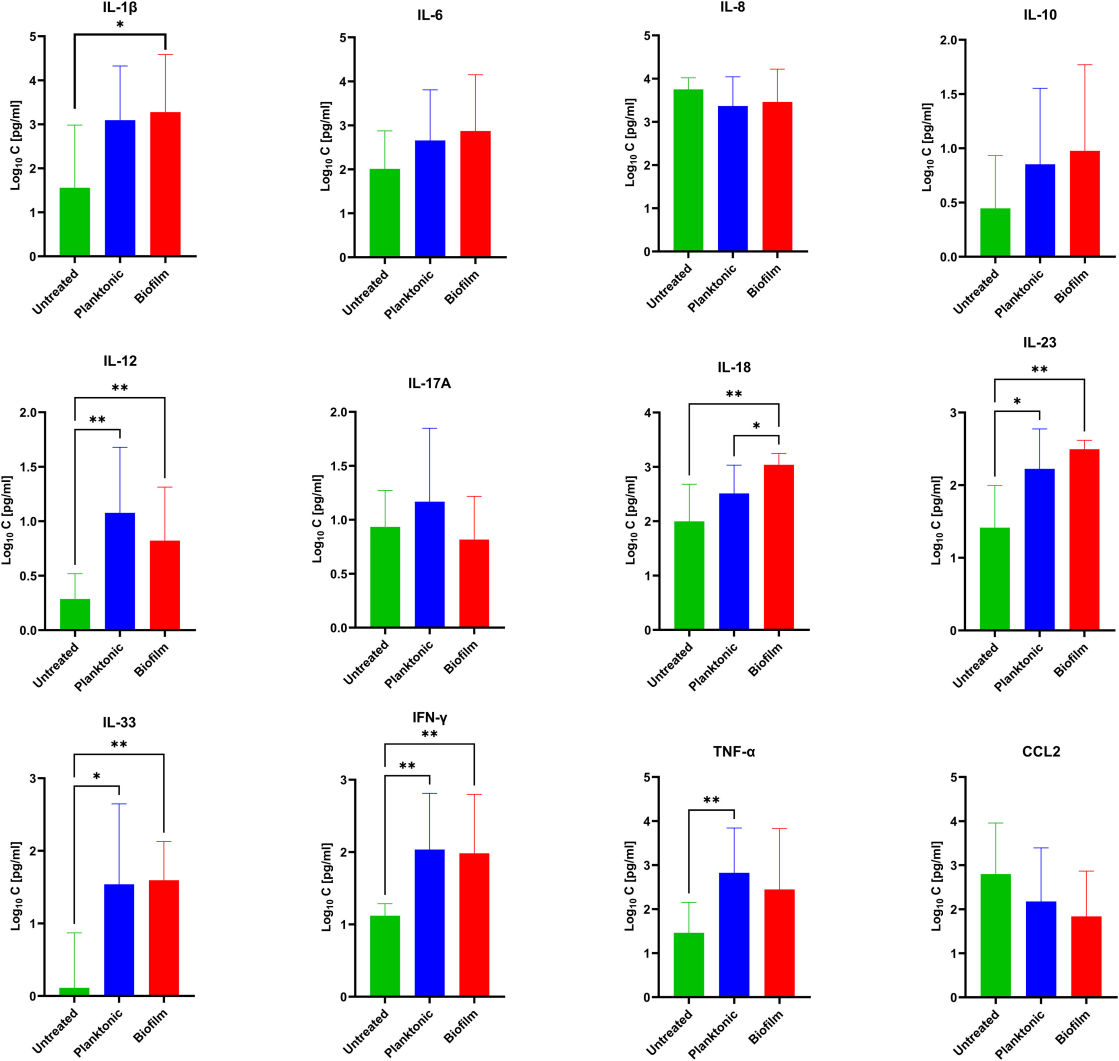
Concentrations of 12 secreted cytokines [pg/mL] evaluated under biofilm or planktonic stress conditions by MRSA compared to untreated PBMCs. The values are presented as means and the standard deviation (SD) of six independent experiments. Asterisks indicate significant differences as BH-corrected *q*-values * ≤ 0.05 and ** ≤ 0.01.

As chronic infections are characterized by persistent exposure to the pathogen, we performed a more detailed statistical analysis on the longer exposure time point (24 h). Both biofilms and planktonic bacteria tended to increase a cluster of cytokines including IL-12, IL-23, IL-33, and IFN-γ, while the levels of IL-6, IL-8, IL-10, IL-17A, and CCL2 were not significantly different (**Figure 3**). Among all cytokines, only TNF-α tended to be elevated in the supernatants of PBMCs co-cultured with the planktonic form of MRSA, while IL-1β tended to be higher under biofilm exposure. Only IL-18 showed significantly increased levels in the supernatant of PBMCs co-cultured with biofilms compared to both the untreated controls and the planktonic MRSA.

The unsupervised hierarchical clustering approach showed that the release of cytokines in response to the stress by biofilms and planktonic MRSA are not clearly differentiable (**Supplementary Figure S2**). Hence, we performed supervised learning and trained machines based on several different concepts (Random Forests, Artificial Neural Networks, and linear discriminant analysis). We compared their predictive power on a randomly selected, unseen test set using 10-fold cross-validation that additionally included one leave-out test set of planktonic or biofilm-challenged PBMCs (two new data sets). Based on the confusion matrix and the area under the receiver operating characteristics curve (AUC_ROC_), the three best-performing machines were artificial neural networks (ANN, AUC_ROC_ = 0.96) > random forest (RF, AUC_ROC_ = 0.93) > linear discrimination regression (LDA, AUC_ROC_ = 0.73) (**Table 1**). Thereby, ANN showed the best accuracy, sensitivity, and specificity of ≥90% in differentiating between planktonic and biofilm stress.

**Table 1:** Performance characteristics of the three best-performing machine learning algorithms.

ANN_CAP_	LDA	RF	ANN	Parameter
0.983	0.73	0.93	0.96	AUC_ROC_ (CI_5-95%_)
0.826	0.63	0.80	0.90	Accuracy
0.90	0.76	0.90	0.90	Sensitivity
0.883	0.50	0.70	0.90	Specificity

ANN, artificial neural network; CAP, community-acquired pneumonia; RF, random forest; LDA, linear discrimination regression; AUC_ROC_, area under the receiver operating characteristic curve; and CI_95%_, confidence intervals.

**Figure 4:**
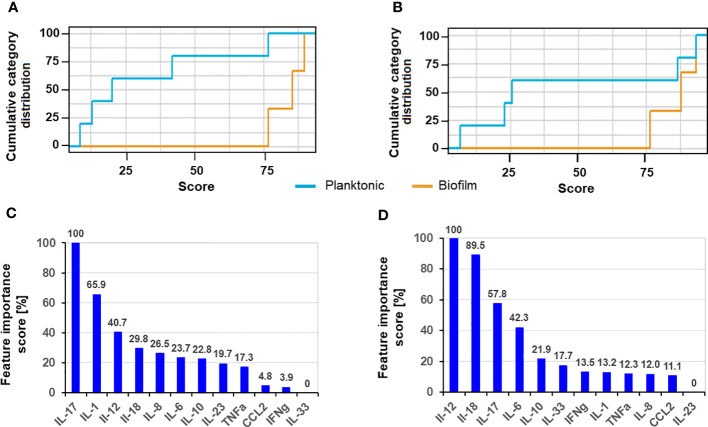
Cumulative category distribution of cytokine concentrations as random variables secreted by PBMC **(A, B)** and artificial neural network feature importance score (expressed in %) **(C, D)** upon exposure of 8 h **(A, C)** and 24 h **(B, D)** to planktonic MRSA or its biofilms.

We used ANN to elucidate the cytokines with the most discriminative secretion pattern due to the different forms of *S. aureus* after 8 h and 24 h of exposure to planktonic and biofilm forms. The cumulative distribution of the cytokine pattern showed a slightly stronger separation of the biofilm and planktonic variables (**Figures 4A, B**) after 24 h exposure. The feature importance scores (IS) for both time points differed slightly, agreeing with the previously described kinetic data, where differentially secreting cytokines show greater differences over time (**Figure 2C**). The cytokines with the highest IS (>60%) after 8 h exposure were IL-17 > IL-1 (**Figure 4C**); in contrast, they were IL-12 > IL18 after 24 h (**Figure 4D**). IL-12 and IL-18 were the third- and fourth-ranked cytokines for the 8 h exposition, but only IL-17 was ranked as third after 24 h exposure; the fourth was IL-10.

### Proteome analysis of PBMCs challenged with biofilms or planktonic bacteria

**Figure 5:**
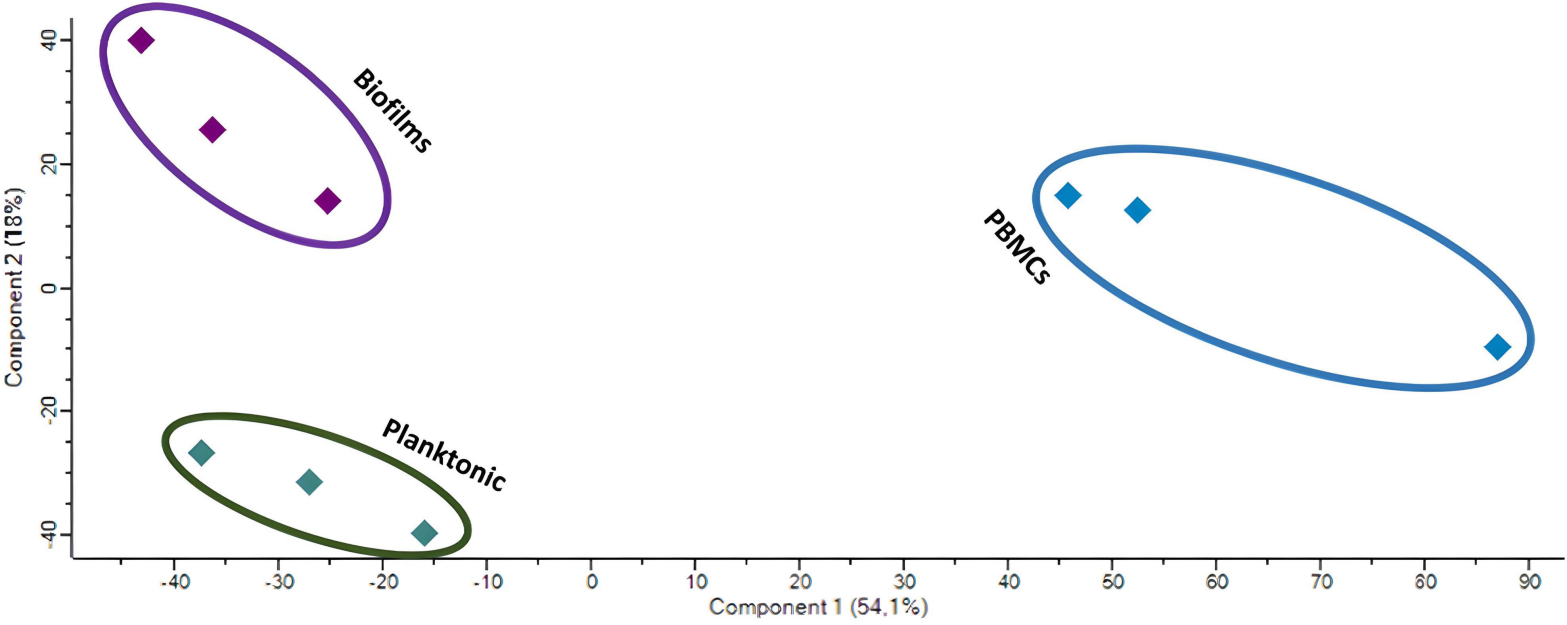
PCA of the LC-MS/MS data of the untreated PBMCs (blue) and those challenged with biofilms (purple) or planktonic (green) *S. aureus*.

To find more differentially secreted proteins and patterns, the secretome of PBMCs challenged with biofilms, planktonic MRSA, or untreated control group was determined by mass spectrometric analysis coupled to liquid chromatography (LC-MS/MS). We identified 1305 ± 227 proteins in the control group, 2643 ± 168 in the planktonic-challenged group, and 1666 ± 162 in the biofilm-challenged group. The principal component analysis (PCA) analysis of the LC-MS/MS data showed a distinct clustering pattern of the repeats among individual treatment groups (**Figure 5**).

**Figure 6:**
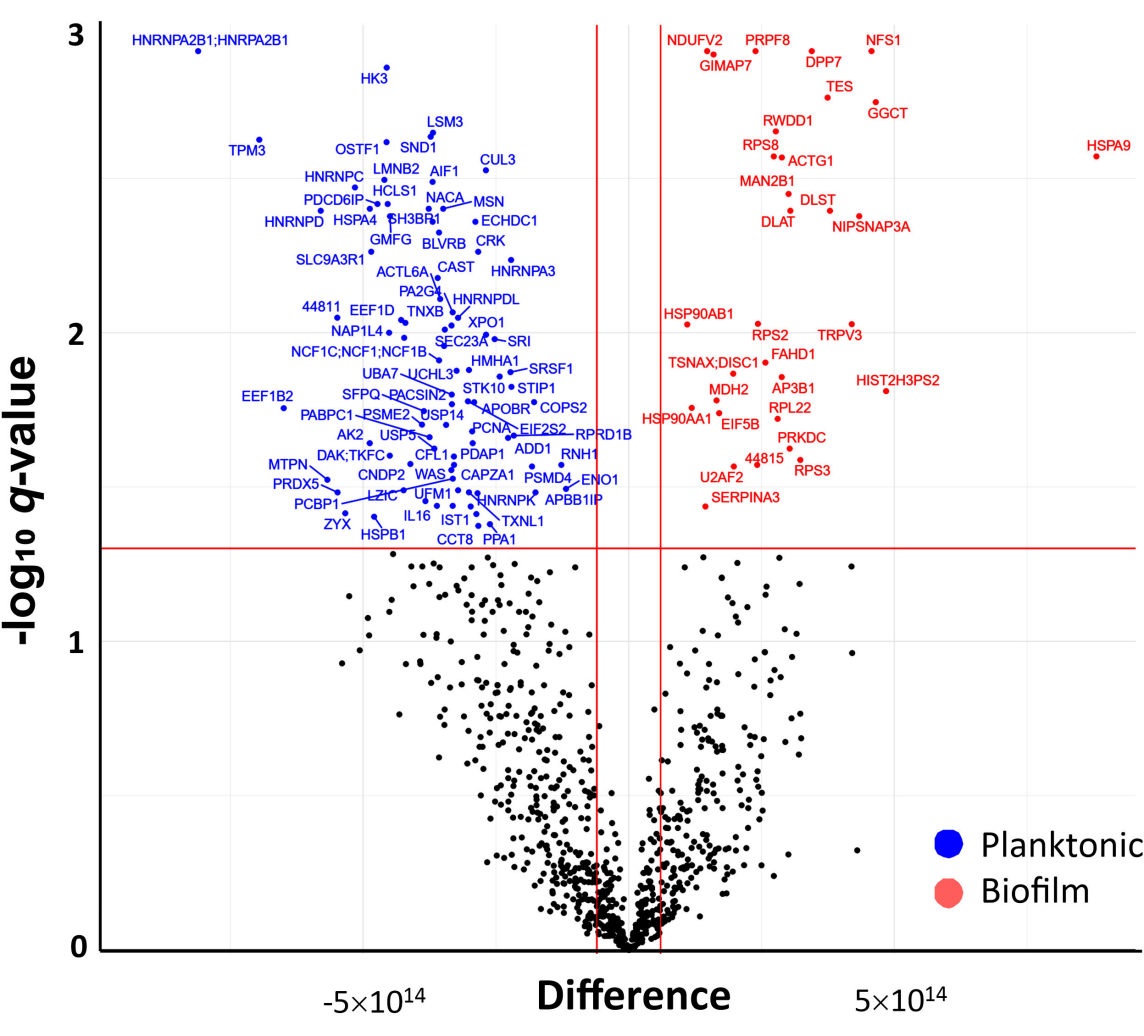
Volcano plot (-log_q_ versus log_FC_) showing the difference in abundance of secreted proteins in PBMC exposed to biofilm in comparison to those exposed to planktonic bacteria. Proteins with a significant abundance change, two-fold higher (log_FC_ > 1) or lower (log_FC_ < -1) secretion (indicated by the vertical red lines), and BH-corrected p-values <0.05 (q-value, indicated by the horizontal red line) are highlighted (blue = higher abundant in planktonic MRSA stimulations and red = higher abundance in biofilm forms stimulations).

The differentially secreted proteins after each treatment were identified via t-test based on Benjamini-Hochberg-adjusted p-value; proteins with q-value < 0.05 and an abundance fold change (FC) > 2 (corresponding to logFC >1) compared to the control group were considered significantly changed in the cell culture supernatants. In PBMCs challenged with MRSA biofilms, 321 proteins were secreted to a higher or lower content, while 298 differentially secreted proteins were identified in PBMCs challenged with planktonic bacteria. Comparing the two challenge conditions, 152 proteins were only detected in supernatants of biofilm-stressed PBMCs, while 129 were only found in supernatants of PBMCs exposed to planktonic forms; in both supernatants, 169 proteins were common. To identify significantly different secreted proteins under both treatment conditions, a t-test was performed and the results were depicted in a volcano plot (**Figure 6**). In total, 84 (blue dots) proteins were identified with a significantly lower abundance and 33 (red dots) to a higher amount when the PBMCs were challenged with biofilms or planktonic MRSA, respectively. (For a detailed list of the proteins, see **Supplementary Table S2**).

**Figure 7:**
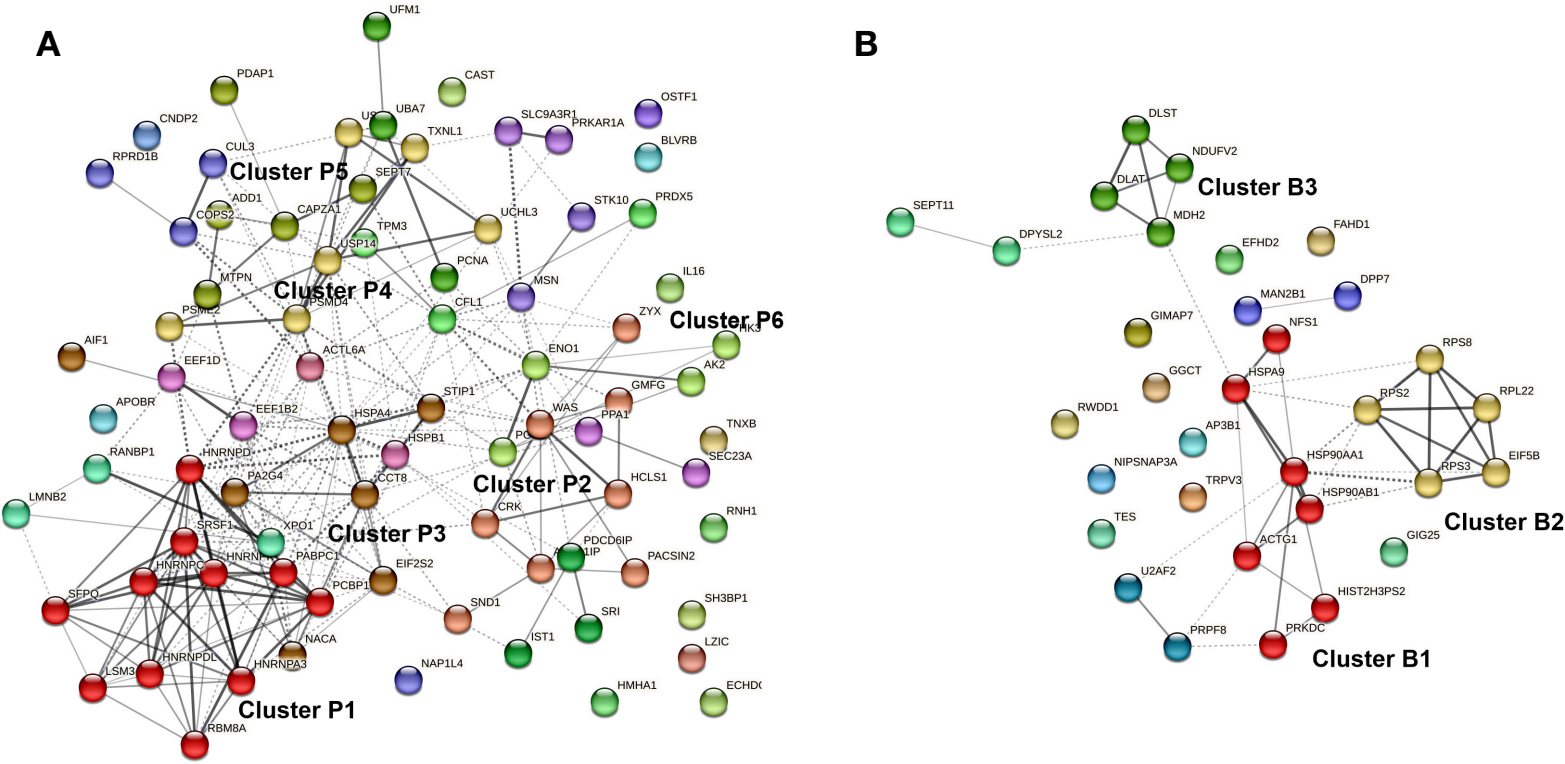
Protein-protein interaction (PPI) network of proteins differentially secreted by PBMCs upon exposure to planktonic *S. aureus* **(A)** or their biofilm **(B)**. The Markov Clustering (MCL) was performed to visualize the most relevant hubs. Only clusters bearing at least four proteins were highlighted. Edges confidence correlates with the strength of the PPI.

The differentially abundant proteins in biofilm or planktonic MRSA stimulations were further investigated based on the corresponding GO terms and visualized as a protein-protein interaction (PPI) network (**Figure 7**). There were 247 edges for the planktonic condition and 44 edges for the biofilm condition. The proteins secreted by PBMCs upon stress with planktonic MRSA formed one major and strongly interacting cluster P1 (red in **Figure 7A**) containing five heterogeneous nuclear ribonucleoproteins and five proteins involved in mRNA splicing, and one protein rather associated with regulation of viral RNA replication and translation (PCBP1). The cluster P2 (salmon) includes proteins involved in the actin cytoskeletal remodeling, cell motility, endocytosis, and staphylococcal-induced gene regulation (SND1). The proteins of cluster P3 (brown) showed weaker interactions and belonged to unrelated pathways. Two of these proteins seem to be more closely related to cluster P1: the cardiac- and muscle-specific transcription factor (NACA) and a proliferation-associated protein (PA2G4) that may be involved in growth and rRNA processing regulation. Further proteins were a chaperon, a stressed-induced co-chaperon, a heat shock protein, and a translation initiation factor 2 subunit, indicating relations to stress response, as well as the allograft inflammatory factor 1 (AIF1) that plays a role in phagocytosis and migration.

Cluster P4 (yellow) contains primary proteasome-associated proteins (such as three ubiquitin carboxyl-terminal hydrolases, subunits proteasome activator, and proteasome non-ATPase). In close connection to cluster P4, there are two ubiquitin-fold modifier proteins and a proliferating cell nuclear antigen (lime green). Cluster P5 (olive) contains proteins associated with cell differentiation and growths (e.g., proteins of cytoskeleton maintenance and growth factors) but also myotrophin that plays a role in the NF-kappa-B circuit. Cluster 6 (green) contains proteins involved in energy homeostasis related to glycolysis and ATP circuits.

For the secreted proteins associated with biofilm stress in PBMCs (**Figure 7B**), three highly connected protein nodes (hubs) (cluster B1, B2, and B3) and three poorly connected hubs (blue, purple, and light green) were identified. Cluster B1 (red) contains three chaperons, one actin (related to motility), and a DNA-dependent protein kinase catalytic subunit required for double-strand break repair and somatic recombination at early stages of T and B cell maturation. The latest seems to be more related to the cellular stress response. The two other proteins were a cysteine desulfurase and a histone-related pseudogene, whose impact on stress response is elusive. Cluster B2 (yellow) is connected to B1 and contains four ribosomal proteins and a translation initiation factor. Cluster B3 (lime green) is related to enzymes involved in the tricarbon acid cycle and thus to the mitochondrial energy process and amino acid precursors.

### IL-16 secretion

Interestingly, the secretome analysis revealed that IL-16 presence was associated with planktonic *S. aureus*. IL-16 was not included in the bead-based cytokine assays performed previously. A separate ELISA for IL-16 was performed showing significant differences in IL-16 levels when compared to the untreated control. However, we could not prove that there was a significant increase in IL-16 secretion in PBMCs when challenged with planktonic *S. aureus* compared to its biofilms (**Supplementary Figure S3**).

### Cytokine profiles of the CAP sera

**Figure 8:**
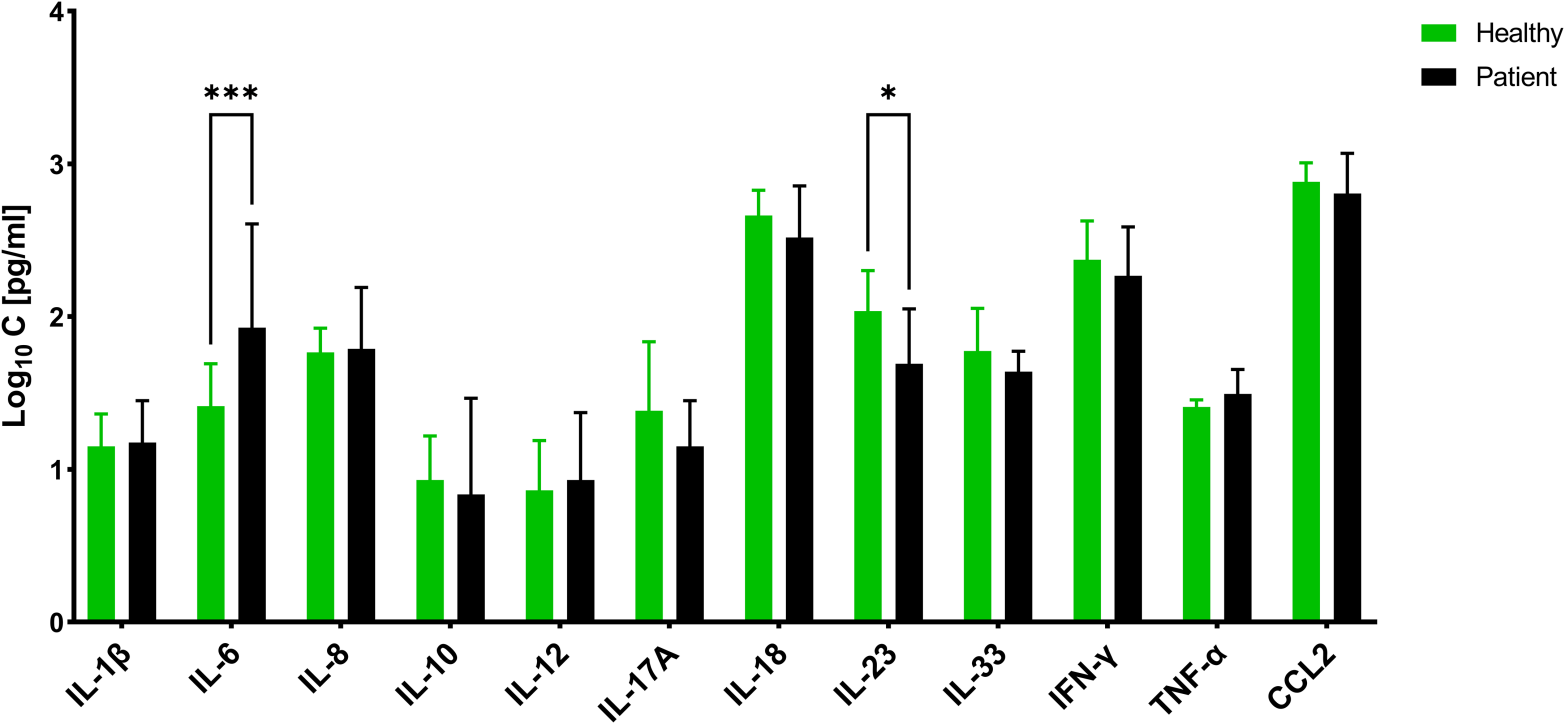
Concentration of different cytokines in Sera of patients suffering from community-acquired *S. aureus* pneumonia and healthy volunteers. The values are presented as means and the standard deviation (SD) of three independent experiments. Asterisks indicate significant differences as BH-corrected q-values * ≤ 0.05 and *** ≤ 0.001.

To assess the concordance between *in vitro* data and patient samples, we conducted an analysis of the cytokine profiles in 30 sera obtained from individual patients diagnosed with CAP caused solely by *S. aureus* (all methicillin-sensitive), as confirmed by approved diagnostic methods (**Supplementary Table S1**). These profiles were compared to those of healthy volunteers. Among the cytokines measured using a multiplex assay, we observed significant differential secretion for only two cytokines (**Figure 8**). Specifically, IL-6 exhibited higher concentrations, while IL-23 exhibited lower concentrations in CAP sera compared to sera from healthy individuals. Interestingly, IL-6 did not show any significant differences in the *in vitro* experiments, but it ranked fourth in terms of importance score after 24 hours of biofilm exposure. On the other hand, IL-23 was found to be expressed at higher levels by PBMCs under both planktonic and biofilm conditions, but it did not receive any importance score.

Subsequently, we aimed to investigate whether the *in vivo* cytokine profiles align more closely with the planktonic or biofilm profile, aiming to gain insights into the extent to which the *in vitro* experiments accurately reflect the immune response in patients. To achieve this, we substituted either the biofilm or planktonic data from the test dataset with the CAP dataset and performed an artificial neural network (ANN) analysis.

**Figure 9:**
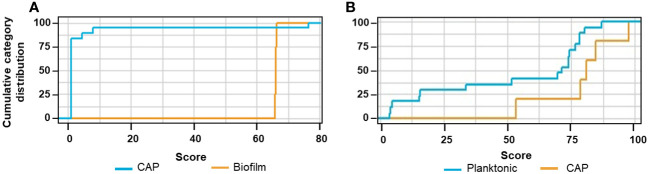
Cumulative category distribution of cytokine concentrations as random variables determined after exchanging the planktonic test data set **(A)** or biofilm test data set **(B)** by then cytokine profiles of the CAP sera.

The cumulative category distribution showed a strong separation of the cytokine profiles of the CAP sera and the biofilm-stressed PBMCs (**Figure 9A**), while separation from the planktonic-induced cytokine profiles of the PBMCs barely split from the CAP cytokine profile (**Figure 9B**).

The ANN clearly differentiated the CAP caused by *S. aureus* from the biofilm stress with an accuracy of 82.6% and an AUC_ROC_ of 0.983; the sensitivity was 0.9 and the specificity was 0.883 (**Table 1**, ANN_CAP_). These predictive parameters were almost as good for the biofilm vs. planktonic data sets indicating that the CAP cytokine profiles were distinct from biofilms.

## Discussion

Chronic *S. aureus* infections such as infective endocarditis (IE) arise from resilient and difficult-to-treat biofilms and result in frequent relapses and complications. The immune response against biofilms is not as effective as against planktonic bacteria. The matrix components and/or other secretory factors produced by the bacteria in biofilms alter the immune response. Several studies have explored the interaction between *S. aureus* biofilms and the immune system; however, limited research has been conducted on human blood cells and living bacteria. Thus, the primary objective of this study was to investigate the *in vitro* immune response induced in PBMCs by planktonic and biofilm-associated MRSA.

The findings of our study showed that *S. aureus* (MRSA) biofilms and planktonic bacteria elicited distinct effects on pro- and anti-inflammatory cytokines secreted by PBMCs. The PBMCs tended to increase secretion of TNF-α when exposed to planktonic MRSA, while exposure to biofilms tended to elevate IL-1β and IL-18 secretion. In the proteome analysis, IL-16 was also differentially highly expressed when PBMCs were stressed with planktonic MRSA but this has not been observed in any biofilm-challenged repeats. However, a separate ELISA showed that both planktonic and biofilms of MRSA resulted in elevated IL-16 levels without significant differences. These results are partially in agreement with a study by Secor et al. who demonstrated strongly increased levels of IL-1β, IL-6, and TNF-α in response to medium conditioned by planktonic *S. aureus* compared to medium conditioned by *S. aureus* biofilms (18). In our study, there was no significant difference in IL-6 secretion in comparison to the uninfected PBMCs. This difference could potentially arise from the variation in cell types exposed to MRSA in our studies. However, we also noted a significantly stronger secretion of most cytokines induced by planktonic bacteria, which became noticeable and persisted after a few hours of stimulation. The biofilms induced cytokine secretion only very weakly, apart from a rapid increase in CCL2 and IL-10 after 8 h of stimulation, which subsequently declined after 24 h. This might be a result of free bacteria detached from the biofilm.

It was previously shown that inactivated Gram-positive bacteria generally stimulated the predominant secretion of IL-12 and INF-γ, while Gram-negatives stimulated the secretion of IL-6, IL-8, and IL-10; the secretion of IL-1β was equally high for both bacteria types (19, 20). These findings corresponded to the planktonic situation in our study. In line with those studies, we did not observe increased levels of IL-6, IL-8, or IL-10 in PBMCs treated with viable biofilms or planktonic MRSA. IL-12 and INF-γ played a critical role in Th1 differentiation. The Th1 activation by planktonic *S. aureus* was understandable, as Th1 cells mediated inflammation *via* the activation of macrophages, B cells, and dendritic cells, but increased cytotoxic activity in macrophages killed intracellular pathogens, and *S. aureus* is known to invade and disseminate *via* macrophages (21).

*In vivo* experiments in mice of catheter-induced *S. aureus* infection showed a significant reduction in cytokine secretion of IL-1β, TNF-α, CXCL2, and CCL2 during biofilm infection (22). In this study, macrophages co-cultured *in vitro* with *S. aureus* biofilms were limited in phagocytosis despite the penetration of the biofilms and showed a gene expression pattern that was more consistent with the M2 macrophages, indicating that *S. aureus* biofilms modulated the immune response toward anti-inflammation (22). Another *in vivo* study examining implant and biofilm-associated *S. aureus* infections in mice demonstrated increased concentrations of Th1-response-related cytokines IL-2, IL-12, TNFα, IL-1β, and the Th17-response-related cytokines IL-6 and IL-17 (most likely IL-17A, but not further specified in the publication) (23). The authors suggested that *S. aureus* biofilms induce early Th1 and Th17 inflammatory responses and downregulate Th2 and Treg responses, which can result in tissue damage, facilitating the formation and maintenance of the biofilm. We did not measure IL-2, but the other cytokines except for IL-17A were generally increased in PBMC secretome upon MRSA exposure. However, in our study, significance was only found for IL-12 independently of planktonic bacteria or biofilm MRSA, and for TNFα under planktonic stress. The differences might be more a result of the *in vitro* setup in our study rather than the resistance phenotype; however, we cannot exclude that the observed effects were influenced by the methicillin resistance-associated penicillin-binding protein (PBP2a). A study with recombinant PBP2a vaccine showed increased IFN-γ, IL-4, and IL-17 cytokines in mice when combined with an adjuvant (24). However, as the vaccine experiments were performed with a high concentration (20 µg per injection) of the PBP2a, the methicillin susceptibility may exert a minor influence on immune cell response. SpA expression levels may influence the cytokine pattern (25), which could explain the differences between our and other studies, but we have not quantified SpA concentrations nor has this been done in the mentioned studies, so we cannot draw a conclusion from this.

Interestingly, IL-12 concentration declined with the infection duration in our study. The level of IL-12 was already high after 2 h and declined during the exposure to the biofilms in contrast to planktonic cells where it showed a time-dependent increase. Since biofilm infections are usually associated with protracted courses and chronification, we believe that the results for 24-hour exposure better reflect the longer exposure of chronic situations *in vivo*. The recursive feature elimination (REF) indicated for the 24h data showed that the cytokines IL-12 (IS = 100%), IL-18 (IS = 89.5%), and IL-17 (IS = 57.8%) were the most important variables in discriminating between the biofilm and planktonic *S. aureus* stress by the machine learning algorithm ANN. Lower importance was assigned to IL-6 (IS = 42.3%) and IL-10 (IS = 21.9%). This was intriguing due to missing significance in the secretion pattern of IL-6 and IL-17, and IL-12 secretion was generally associated with *S. aureus*. This illustrates how data can be interpreted differently when various analytical methods are employed.

In fact, it is challenging to utilize this for diagnostic purposes since only individual samples are measured, and one must be able to infer from the current data if a biofilm is the underlying source of infection, as this will be decisive for more aggressive antimicrobial treatment. Based on the results, no specific marker can be derived that differentiates between an acute infection/inflammation, which primarily indicates the presence of planktonic bacteria in the blood and a chronic biofilm-associated infection; instead, a panel of cytokines may provide a more nuanced representation of the infection status. The most potent marker, which might indicate the presence of a biofilm, would indeed be IL-18. Interestingly, so far, IL-18 has not been correlated to *S. aureus* biofilms, as it was not covered by the *in vitro* or *in vivo* studies performed so far.

IL-18 is a proinflammatory cytokine that is secreted by macrophages and MAIT cells in response to viral and bacterial infections, but its overproduction has been associated with autoimmune diseases (26). The role of IL-18 in *S. aureus* infection is not yet fully understood, but it was shown in an atopic dermatitis (AD) mouse model that *S. aureus* increases the epidermal IL-18 production that was dependent on the activation of the NLRP3 inflammasome (27, 28). IL-18-deficient mice exhibited reduced skin inflammation compared to wild-type mice, which was associated with decreased expression of proinflammatory cytokines such as IL-1β, TNF-α, and IL-6 (28). Increased levels of IL-18 were also confirmed in the stratum corneum of lesional skin of AD patients, particularly those colonized with *S. aureus* (27). These studies and our results suggest that IL-18 may be generally associated with *S. aureus* colonization and biofilm formation.

Capturing a signature of immune cells’ interaction with MRSA biofilm infections might help in finding more potential biomarkers; we identified 156 proteins being differentially secreted by PBMCs under biofilm conditions by proteome analysis. Within those, we identified IL-16, which however did not prove to be discriminative for biofilms in the ELISA assays as it was generally increased upon MRSA exposure. The proteomics, however, demonstrated that the secretome of PBMCs splits into distinct clusters in the PCA, indicating that there might still be potential well-discriminating protein factors that could prove as markers for biofilm infections caused by MRSA. The PPI network showed that the proteins particularly related to biofilm stress on PBMCs cover proteins involved in general protein circuit (like chaperons), DNA repair, cell growth, and energy circuit, while planktonic stress-induced proteins involved in mRNA splicing, cell plasticity, and proliferation, as well as motility and phagocytosis. All this indicates that planktonic MRSA primarily induces pro-inflammatory processes in the cell, which is related to the mobilization of the immune cells, adaptation of the receptors and antibodies to the pathogen, phagocytosis, and finally the release of corresponding pro-inflammatory cytokines, which were also measured in this study. The biofilms tended to promote significantly less immune-stimulating processes related to more ordinary cell growth and maintenance of cell functions. This supports the hypothesis that biofilms are more likely to promote the anti-inflammatory response in immune cells.

From our point of view, it will hardly be possible to determine a specific factor that will be discriminatory for *S. aureus* biofilm infections. Possibly, a ratio score of IL-1β and IL-18 might be more suitable, as both were predominantly secreted under planktonic or biofilm conditions, respectively, and might be therefore more reliable. However, investigations with different and larger cohorts are needed to demonstrate the reliability of this index for clinical use. On the other hand, the question arises: can certain infections be assigned to a certain form of *S. aureus in vivo*? In the case of biofilms, it appears to be a little more effective, but can one really call an acute infection planktonic?

To do this, we labeled the measured cytokines of CAP sera, which are acutely ill from a clinical standpoint, as either planktonic or biofilm and used as a test set in the ANN algorithm to see whether such an assignment would be appropriate for patient samples in a diagnostic setup. The cytokine profile was distinct from both biofilm and planktonic-stressed PBMCs, but the IL-6 and IL-23 were also present in significantly higher concentrations in CAP sera compared to sera of healthy individuals. The machine learning results were astonishing as the cytokine patterns stimulated by *in vitro* biofilms were clearly distinguished from the sera obtained from CAP patients, in contrast to those *in vitro* data stimulated by planktonic forms. Remarkably, the predictive power and accuracy were nearly as good when comparing the stimulation of PBMCs by planktonic and biofilm forms of *S. aureus*. However, this does not exclude the possibility of biofilm formation in CAP patients since the samples were collected within 48 h of hospitalization.

Our examination was limited to the sera samples, which showed a reduced tendency of cytokine secretion. Individual cytokine levels have not been shown to differ significantly between healthy controls and patients suffering from lung infections such as bronchiectasis. In the study by Ayhan et al., there were differences only for IL8 and IL10, and these were found in both serum and bronchoalveolar lavage (BAL) (29). In the study by de Brito et al., in different pediatric pneumonia of 11 specific cytokines, only IL-10 and IL-6 were discriminative for mild and severe pneumonia (30). Thus, it appears that plasma levels of most cytokines generally differ greatly in pneumonia. The fact that other cytokines tended to show differences in CAP than in bronchiectasis could be due to the entity but also generally to a high level of variability between patients.

We also need to consider that tissue-resident immune cells must first respond to *S. aureus*. Then the immune signature would be translocated to the bloodstream. Future research should aim to determine if it is possible to identify the ‘silent’ formation of chronic infections. These infections are characterized by biofilms that remain inactive within organs in patients. However, this will be a more challenging endeavor as most patients admitted to the hospital suffer from acute symptoms, where the acute presence of bacteria in the bloodstream can be assumed. Designing a study with clinical samples to differentiate biofilm-associated infections early in the process of chronification due to biofilm establishment represents the next milestone in our research efforts.

### Limitations of study

The main limitations of this study are it is an *in vitro* design using PBMCs and only one strain is used with a specific phenotype (MRSA). The use of PBMCs has advantages and disadvantages. One disadvantage is that we did not perform measurements on isolated immune cell subtypes, which would have provided insights into the specific signaling in selected cells stimulated by biofilms or planktonic *S. aureus*. On the other hand, this would also have its drawbacks as the immune response is not solely the result of individual immune cell subtypes but rather the interplay of all immune cells and plasma components. Therefore, we do not consider the use of PBMCs as a disadvantage but rather as an opportunity to gain more insights into the immune response. However, it should be noted that *in vitro* conditions often do not fully replicate the *in vivo* studies due to various factors. Consequently, future studies need to include appropriate patient cohorts to better understand the interaction of *S. aureus* biofilms with the immune response.

The use of only one MRSA strain could distort the results. *S. aureus* can express different virulence factors, which can influence the immune response differently. Methicillin-sensitive *S. aureus* (MSSA) is generally more virulent than MRSA, which suffers some loss of fitness due to its altered resistance factors. This has been shown in some animal studies. In an intradermal infection mice model, IL-1β, IL-6, TNF-α, and IFN-γ were produced by the lymph nodes only after MSSA infection; while in a murine model of endophthalmitis, IL-10, IL-15, IL-11, and some chemokines and receptors were significantly strongly upregulated by MRSA (31). This means that the differences could also depend on the location of the infection. Unfortunately, comparable study data from infections with MRSA and MSSA in humans are not available. Despite this possible variance, we primarily examined the differences between planktonic and biofilm forms in the present study. Therefore, from our point of view, it seems more expedient at this point to simplify the model and only consider one laboratory strain. Admittedly, the transfer of experimental results and evaluation to CAP sera could be biased by the phenotype of *S. aureus*. We do not have information on the phenotypes of the CAP *S. aureus* isolates, but we assume that the CAP isolates were rather MSSA since MRSA rarely occurs in community-acquired infections in Germany. However, the algorithm strictly differentiated the CAP sera from the biofilm type, which in our opinion supports the robustness of the machine-learning assignment.

## Methods

### Resource availability

#### Lead contact

Further information and requests for resources and reagents should be directed to and will be fulfilled by the Lead Contact, Reza Gheitasi (reza.gheitasi@med.uni-jena.de).

### Method details

#### Storage and culturing of bacterial strains

The reference strain methicillin-resistant *S. aureus* (MRSA) ATCC 43300 was purchased from the American Type Culture Collection, Manassas, USA. This bacterial strain is *pvl* negative and *SCCmec* type II, not superantigen secretor, and it is resistant to methicillin and oxacillin (32). Stock cultures were established by cultivating bacteria in tryptic soy broth (TSB) (Merck KGaA, Darmstadt, Germany) overnight. Bacterial suspensions were then aliquoted and stored in the same medium supplemented with 10% glycerin at -80°C. For the experiments, the bacteria were freshly streaked from the stocks on blood agar plates (Merck KGaA, Darmstadt, Germany) and incubated overnight at 37°C. Fresh TSB culture was prepared by resolving a single colony in 10 mL broth and overnight incubation with shaking (150 rpm) at 37°C.

#### Isolation of peripheral blood mononuclear cells

Peripheral blood from six anonymized healthy volunteers was purchased from Institut für Klinische Transfusionsmedizin Jena GmbH (Jena, Germany). Peripheral blood mononuclear cells (PBMCs) were isolated using a gradient procedure as described previously. Briefly, blood collected in 10 mL EDTA K3 tubes (S-Monovette^®^, Nümbrecht, Germany) was 1:1 diluted with PBS in a 50 mL tube (Falcon Corning, NY, USA). The mixture was layered with the lymphouprep™ density gradient medium (Stemcell Technologies Inc., Vancouver, Canada) and centrifuged at 1200 g for 15 min at room temperature (RT) according to the manufacturer’s protocol. After centrifugation, PBMC layers were transferred into a new conical tube, washed three times with 5 mL PBS containing 2% heat-inactivated fetal bovine serum (FBS), and finally resuspended in an enriched RPMI medium (if needed supplemented with 10% FBS and 2 mM L-glutamine) (all Thermo Fisher Scientific, Waltham, USA). The viable cells were counted after staining with 0.4% trypan blue solution (Thermo Fisher Scientific) using countees III device (Thermo Fisher Scientific).

#### Serum from community-acquired pneumonia patients

A set of 30 serum samples from patients with community-acquired pneumonia (CAP) with confirmed *S. aureus* diagnosis without other co-infections were obtained from the CAPNETZ Stiftung (Hannover, Germany) for cytokine profiling. CAPNETZ Stiftung collects materials and data from CAP patients throughout Germany. Sera were collected from CAP patients within 48 h after hospitalization. Corresponding ethics votes and written patients’ consents are available in the respective institutions, which enabled the material to be passed on to research institutions for scientific purposes upon positive approval of a research application (project number J8JD) by CAPNETZ Stiftung. Information on some patients’ parameters is provided in **Supplementary Table S1**.

#### Biofilm formation in RPMI medium

From fresh TSB overnight cultures, a volume of 0.5 mL was centrifuged at 3500 g at RT for 5 minutes and the pellet was resuspended and adjusted in enriched RPMI medium (contains 10% FBS, 2 µM L-glutamine) to an optical density of 0.07 at 600 nm (OD_600_). The colony-forming units (CFU) per mL in the inoculum suspensions were confirmed by log-microdilution and colony counting on TSB agar plates after incubation overnight at 37°C. For biofilm formation, 200 µL of the bacterial suspensions were applied per well into 96 flat-bottom wells plate (Greiner Bio-One GmbH, Frickenhausen, Germany) in triplicates and incubated for 24 h at 37°C without shaking in humidified condition with 5% CO_2_. The biofilms were gently washed three times with pre-warm phosphate-buffered saline (PBS) (Carl Roth GmbH, Karlsruhe, Germany) to remove free planktonic cells and subsequently exposed to different treatments as described below.

#### Confocal laser scanning microscopy

The washed biofilms were fluorescently stained using the LIVE/DEAD BacLight Bacterial Viability Kit for microscopy (Life Technologies GmbH, Darmstadt, Germany) according to the manufacturer’s protocol. The SYTO9^®^ stains all bacteria in green and the propidium iodide counterstains the dead bacteria in red. The stained samples were analyzed under vital conditions using an inverse confocal laser scanning microscope equipped with an argon laser line (CLSM) LSM780 and images were processed using ZEN Black software (both Carl Zeiss AG, Jena, Thuringia, Germany). The CLSM images were performed by applying excitation at 490 nm and using the 40x air objective. An area of approximately 100 μm (X-dimension) x 100 μm (Y-dimension) was screened in 1 μm Z-intervals (Z-stack) at green (522 nm) and red (635 nm) channels, respectively. The pinhole was adjusted to 1 μm.

#### Challenging the PBMCs with *S. aureus*

The PBMC aliquots were resuspended in enriched RPMI and 200 µl of the suspension containing 0.5 x 10^6^ cells/well were added to the 24 h old *S. aureus* biofilms (1 x 10^7^ cells/well) or mixed with 200 µl of 1 x 10^7^ CFU/ml *S. aureus* suspension. These co-cultures were incubated at 37°C and 5% CO_2_ for 24 h. As a negative control, the PBMCs alone were cultured under identical conditions. As an additional control, the PBMCs were stimulated with 5 μg/ml phytohemagglutinin (PHA-L) (Thermo Fisher Scientific), where increased production of most of the measured cytokines could be observed confirming the viability of the PBMCs (**Supplementary Figure S3**). Following incubation, the PBMCs’ viability was analyzed by trypan blue staining (see above) from 50 µl aliquots. The majority of the PBMCs (75.5% ± 7.04%) remained viable following incubation with pathogens. The supernatants were collected by centrifugation at 500 g for 5 min at RT, sterile filtered (0.22 µm pore size), aliquoted, and stored at -80°C until further tests (i.e., cytokine determination).

#### Cytometric bead array

The levels of a panel of cytokines were determined in the supernatants of the PBMC from healthy individuals that were co-cultured with 24-h-old *S. aureus* biofilms and planktonic *S. aureus* cells, as well as serum samples from CAPNETZ patients. The bead-based multiplex assay Legendplex™ Human Inflammation Panel 1 (BioLegend, San Diego, USA) was used according to the manufacturer’s instructions. We included the following cytokines: IL-1β, IFN-γ, TNF-α, CCL2, IL-6, CXCL8, IL-10, IL-12p70, IL17-A, IL-18, IL-23, and IL-33. IFN-α2 was not included as it is predominantly related to viral infections (33). Samples were measured by Accuri 6 flow cytometer (BD Biosciences, New Jersey, USA), analyzed with LegendPlex online server (Biolegend), and compared to standard curves. The experiments were performed in technical duplicates and six independent biological replicates.

#### Enzyme-linked immunosorbent assay

The level of IL-16 cytokine was determined for the supernatants of different co-cultures using a Human IL-16 ELISA kit (Invitrogen, Thermo Fisher Scientific) following the manufacturer’s instructions. The absorbance at 450 nm of the samples and the standard provided with the kit was measured using the Infinite 200 PRO plate reader (Tecan Trading AG, Männedorf, Switzerland). The concentration of IL-16 was calculated based on the fitting of the standard curve.

#### Training of the machine-learning models

Unsupervised hierarchical clustering and various machine-learning classifiers included in the caret package (34) in R (i.e., artificial neural network (ANN), with two hidden layers), random forest (RF), linear discrimination regression (LDA), support vector machine (SVM), K-nearest neighbor (KNN), and decision tree) were compared. For training the machine-learning algorithms, we used the dataset of 12 different cytokines secreted by PBMCs challenged both by biofilms and planktonic *S. aureus* and measured at different time points (2, 4, 8, and 24 hours) by flow cytometry. The data were normalized using the log2 method due to the high dynamic range of cytokine levels to improve reliability and predictive accuracy. All classifiers were applied on the 24 h values to evaluate which one performs best in distinguishing between biofilms and planktonic stage. The biological replicates were randomly split into one testing replicate (16.6%) and five training replicate (83.3%) sets. The training set was further split into 10 blocks and 10-fold cross-validations were performed permutating the blocks (nine for training and one for validation). The predictive performance of all models was finally assessed using the testing set consisting of the residual replicate and two others so far not analyzed data sets from the biofilm challenge for 24 h. The receiver operating characteristic curve (AUC_ROC_) was calculated, and sensitivity, specificity, and balanced accuracy were assessed based on a confusion matrix. To predict the most discriminative cytokines, recursive feature elimination (RFE) (34) was used for feature reduction and selection.

The best-performing classifiers ANN were also applied to the cytokine profiles determined in the patients’ sera from the CAPNETZ cohort. Once again the ANN algorithm has been trained with obtained *in vitro* data. For the testing of data set B, the planktonic data were replaced by the CAPNETZ data. The AUC_ROC_ and confusion matrix were calculated.

#### Mass spectrometric analysis

Supernatants of the PBMCs and PBMCs challenged with planktonic bacteria and biofilm were supplemented with sodium dodecyl sulfate (SDS) and dithiothreitol (DTT) (end concentrations 1% and 50 mM, respectively) and heated for 5 min at 95°C, and then further diluted in urea buffer (8 M urea, 100 mM Tris HCL, pH 8.0). Buffer exchange and protein digestion was done as follows: The reduced proteins were transferred to a 10 kDa Microcon YM-10 filter (Merck KGaA, Darmstadt, Germany) and centrifuged at 14.000 x g for 20 min in all consecutive steps, and the flow-through was discarded. For washing, 200 µL urea buffer was added and the centrifugation was repeated. A measurement of 100 µL of alkylation solution (0.1 M iodoacetamide in urea buffer) was added and samples were incubated for 20 min in the dark. The alkylation solution was removed by centrifugation followed by two additional centrifugation steps with 200 µL 8 M urea buffer. Afterward, samples were washed and centrifuged twice with 200 µL 50 mM ammonium bicarbonate buffer. Proteins were digested by the addition of 0.5 µg trypsin in 50 µL 50 mM ammonium bicarbonate (all Merck). Proteolytic cleavage was allowed for 16 h at 37°C and peptides were eluted by centrifugation at 14000 g for 20 minutes. To collect residual peptides, the centrifugation was repeated twice after the addition of 50 µL ammonium bicarbonate buffer. Eluted peptides were dried in a SpeedVac (Thermo Fisher Scientific) and reconstituted by adding 25 µL of 0.3% formic acid in water.

Tryptic peptides were analyzed with a Dionex UHPLC coupled to an Orbitrap Fusion LC-MS/MS system (all Thermo Fisher Scientific). Full mass spectrometry scans were acquired in the Orbitrap (m/z range 370-1570, quadrupole isolation) at a resolution of 120,000 (full width at half maximum) within 150 min of a non-linear gradient from 2% to 90% acetonitrile/0.1% formic acid (Merck, USA). Ions were fragmented by higher-energy collisional dissociation (HCD, 30% collision energy) and a maximum of 20 fragment ion spectra were acquired per cycle in the ion trap at rapid scan mode. The following conditions were used: spray voltage of 2. kV, heated capillary temperature of 275°C, S-lens RF level of 60%, a maximum automatic gain control (AGC) value of 4x10^5^ counts for MS1 with a maximum ion injection time of 50 ms, and a maximum AGC value of 1x10^4^ for MS2, with a maximum ion accumulation time of 35 ms. A dynamic mass exclusion time window of 60 s was set with a 10 ppm maximum mass window.

These experiments were performed on three individual samples from healthy volunteers in an independent manner.

#### Protein identification and quantification

All raw files were searched against the human UniProt database (version 05.2016, reviewed sequences), and the uniparc proteome UP000244076 (*S. aureus* strain ATCC 43300), with MaxQuant version 1.6.17.0 (Max Planck Institute of Biochemistry, Germany). Parameters were used or set as follows: first search peptide tolerance: 20 ppm, main search peptide tolerance: 4.5 ppm (for MaxQuant); enzyme: trypsin, maximum 2 missed cleavages; static modification: carbamidomethylation of cysteine residues; variable modifications: methionine oxidation; minimum peptide length: 6, maximum peptide mass: 7600 Da. Normalization was done in MaxQuant using label-free quantification (LFQ), setting the minimum ratio count to two (unique and razor peptides). Further analysis of LFQ and protein intensities was performed using the Perseus software package version 1.6.2.2 (Max Planck Institute of Biochemistry). The LFQ intensities were log2-transformed and missing values were imputed from the normal distribution of the data set (width: 0.3, downshift 1.8). Known contaminants, reverse-identified proteins, and proteins with “identified by site” were discarded. Proteins with less than two identifications in at least one group were removed from the data set.

#### Statistical analyses

The visualization and statistical analysis of the cytokine secretion data obtained by the bead arrays and the ELISA were performed in GraphPad Prism 9 (GraphPad Software, San Diego, California, USA). The One-Way-ANOVA corrected by Benjamini and Hochberg (BH), which allows a better fall discovery rate (FRD) control, was applied, and adjusted *p*-values (*q*) < 0.05 were considered significant.

Differences in the expression of the proteins assessed by LC-MS/MS were analyzed in the Perseus application (35) using a *t*-test with BH correction. Statistical differences in the protein LFQ abundances were assumed for *q*-values <0.05 (BH-adjusted *p*-value). To reduce data dimension, a principal component analysis (PCA) was applied to BH-corrected LFQ values using the Perseus software package (see above). To compare the differences in the secretome among the control groups, the - log10*_q_* values were plotted (volcano plot (36)) against the fold change (FC) in LFQ abundance as log_FC_. Significantly different secreted proteins were assumed at - log10*_q_* > 1.3 (corresponding to *q <*0.05) and log_FC_ > 2 (corresponding to FC < 2). We used the STRING database (Search Tool for the Retrieval of Interacting Genes/Proteins) to visualize the association protein-protein interaction (PPI) network and the Markov Cluster Algorithm (MCL) clustering option (provided by STRING) to identify functional clusters. The functional relation of the proteins was assessed based on the Gene Ontology terms (GO). Here, only the significantly differentially secreted proteins related to biofilm or planktonic stress were utilized.

## Data availability statement

The mass spectrometry proteomics data have been deposited tothe ProteomeXchange Consortium via the PRIDE (37) partnerrepository with the dataset identifier PXD044120. The underlyingcode for this study is available in GitHub and can be accessed viathis link https://github.com/GheitasiR/Nuance-from-S.-aureusplanktonic-to-biofilms.git.

## Ethics statement

The studies involving humans were approved by Jena University Hospital, Jena, Germany. The studies were conducted in accordance with the local legislation and institutional requirements. The participants provided their written informed consent to participate in this study.

## Author contributions

RG: Conceptualization, Data curation, Formal analysis, Investigation, Methodology, Visualization, Writing – original draft, Writing – review & editing. DR: Conceptualization, Writing – original draft. MM: Methodology, Writing – original draft. MN: Formal analysis, Software, Validation, Writing – original draft. RK: Data curation, Formal analysis, Validation, Writing – review & editing. HS: Methodology, Writing – review & editing. MP: Funding acquisition, Project administration, Resources, Supervision, Validation, Writing – review & editing. OM: Conceptualization, Data curation, Project administration, Supervision, Validation, Visualization, Writing – original draft, Writing – review & editing.
