# Supplementary material for: Exploring secretory proteome and cytokine kinetic of human peripheral blood mononuclear cells exposed to methicillin-resistant Staphylococcus aureus biofilms and planktonic bacteria
Source: Front Immunol. 2024 Mar 20;15:1334616. doi: 10.3389/fimmu.2024.1334616 (PMC10989517; doi:10.3389/fimmu.2024.1334616)
Supplement: Supplementary file 1 [file DataSheet_1.docx]

**Exploring Secretory Proteome and Cytokine kinetic of Human Peripheral Blood Mononuclear Cells Exposed to *Staphylococcus aureus* Biofilms and Planktonic Bacteria**

Reza Gheitasi^1*^, Daniela Röll^1^, Mario M. Müller^2,5^, Mohadeseh Naseri^1^, Rainer König^1,5^, Hortense Slevogt^3,4^, Mathias W. Pletz^1,5,6^ and Oliwia Makarewicz^1,5^

^1^ Institute of Infectious Diseases and Infection Control, Jena University Hospital / Friedrich Schiller University, Jena, 07747, Germany.

^2^ Septomics Research Center, Jena University Hospital, Jena, 07747, Germany.

^3^ Respiratory Infection Dynamics, Helmholtz Centre for Infection Research-HZI Braunschweig, 38124 Braunschweig, Germany.

^4^ Department of Respiratory Medicine and Infectious Diseases, Hannover Medical School, German Center for Lung Research (DZL), BREATH, 30625 Hannover, Germany.

^5^ Integrated Research and Treatment Center - Center for Sepsis Control and Care (CSCC), Jena University Hospital, Jena, 07747, Germany.

^6^ CAPNETZ STIFTUNG, Hannover, 30625, Germany

**Running title**: Impact of *S. aureus* biofilms on PBMCs

***Corresponding author**: reza.gheitasi@med.uni-jena.de

**Keywords:** Chronic infections, interleukins, immune response, proteomics, secretome


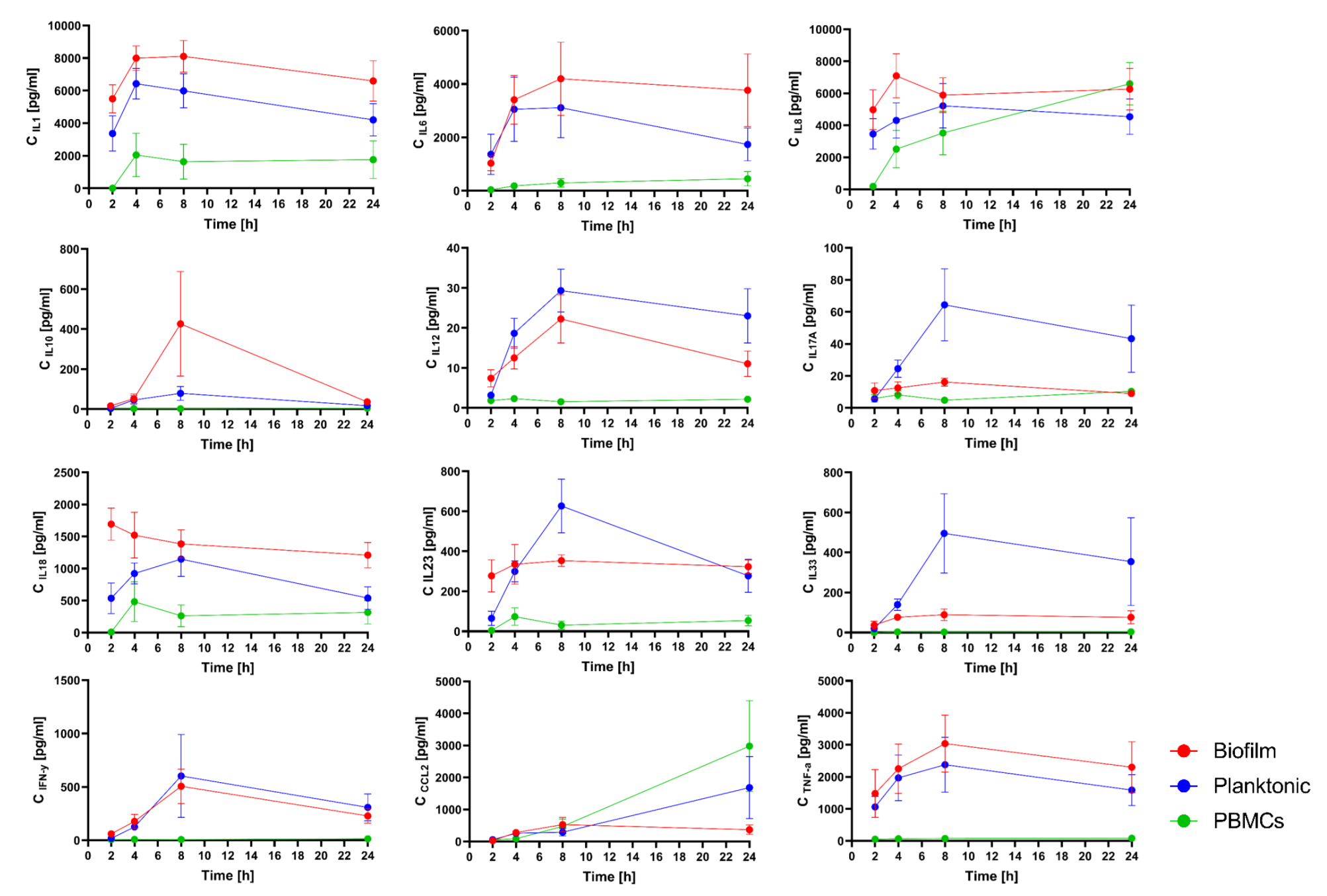


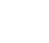

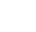

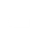

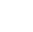

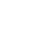

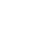

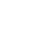

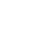

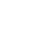


**Figure S1:** Time dependent concentrations of all individual secreted cytokines [pg/mL] evaluated under biofilm (red) or planktonic (blue) challenge by MRSA compared to untreated PBMCs (green). The values are presented as means and the standard deviation (SD).


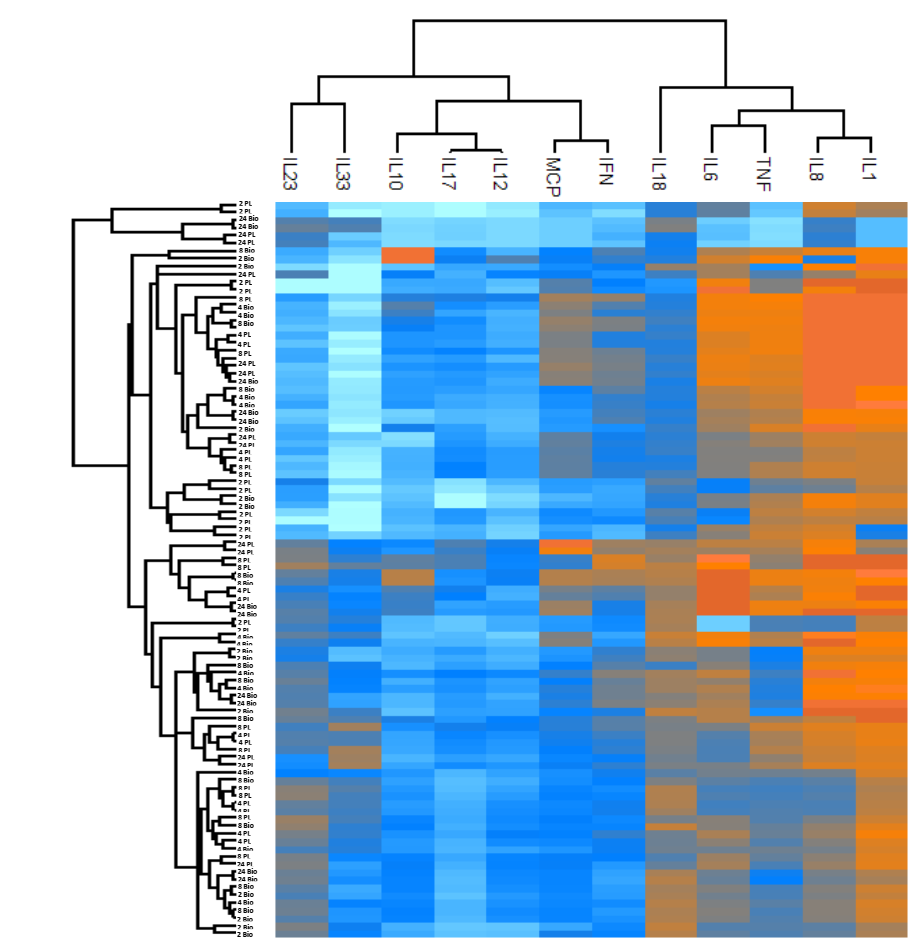


**Figure S2:** Heatmap, displaying unsupervised hierarchical clustering based on different cytokines in response to infection by biofilms (indicated by Bio) or planktonic (indicated by Pl) forms of *S. aureus* bacteria. Samples were taken after 2 h, 4 h, 8 h and 24 h and two replicates are shown that in most cases have been merged with the highest similarity on the left-hand dendogram.


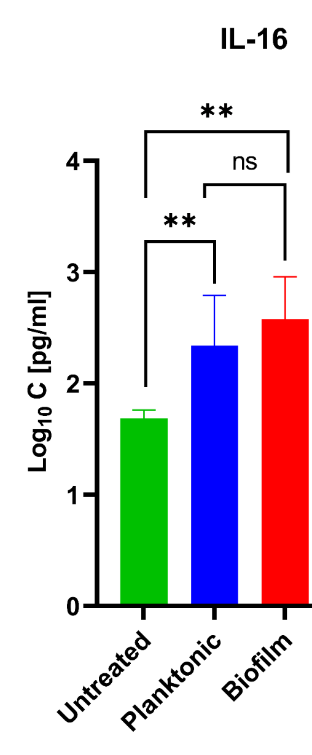


**Figure S3:** The level of IL-16 cytokine [pg/mL] evaluated under biofilm, or planktonic infected PBMCs compared to untreated PBMCs.


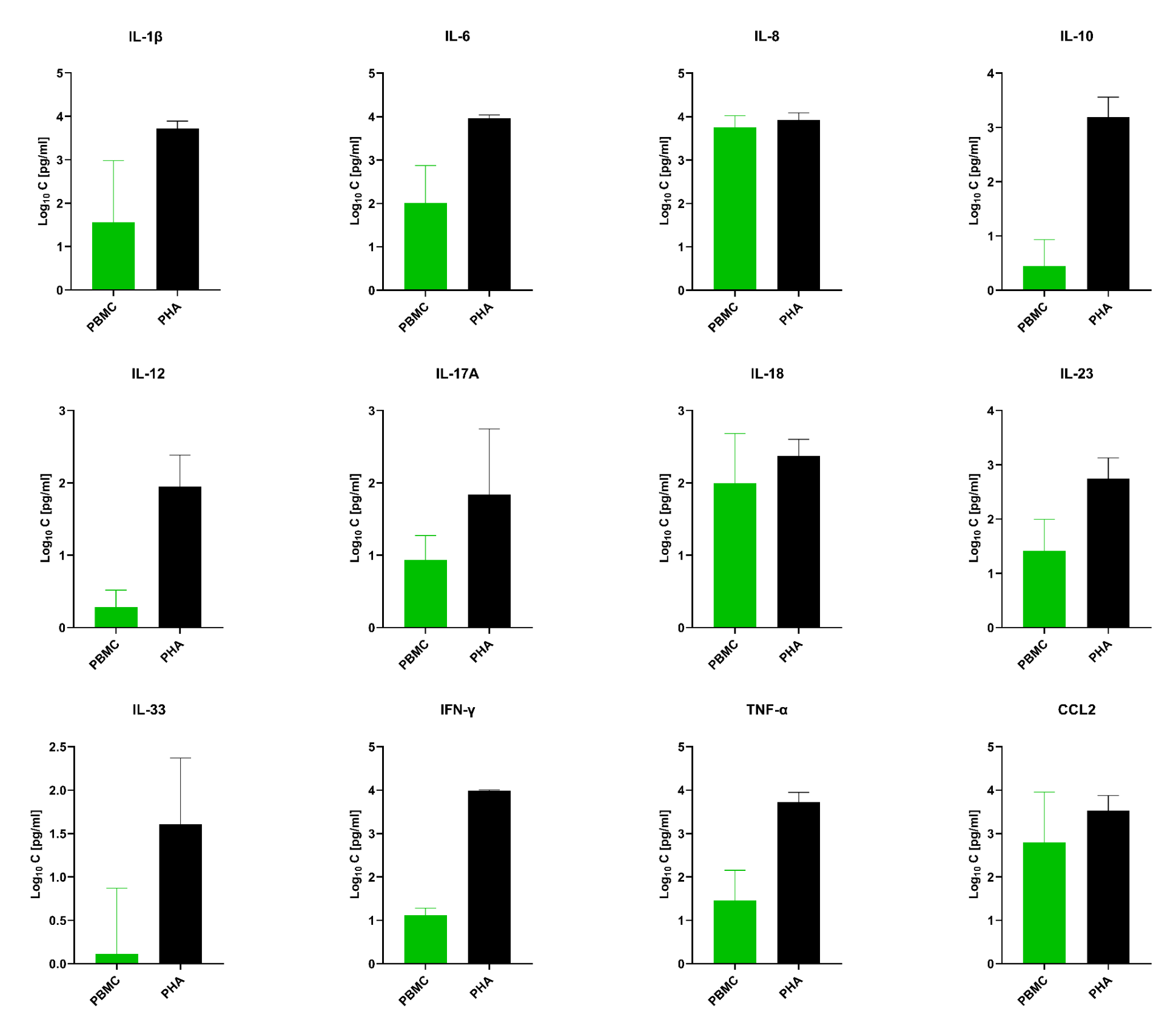


**Figure S4:** Concentration of 12 secreted cytokines [pg/mL] evaluated under 5 μg/ml phytohemagglutinin (PHA-L) stimulation condition and untreated PBMCs.

**Table S1:** Parameters of eh CAP cohort.

| ID | **Sample year** | **Sex** | **Age** | **Antimicrobial treatment in h (before sampling)** | **Puls (per min)** | **Leucocytes (10^9^ per L)** | **Hemoglobin (g/dL)** | **Hematocrit (%)** | **Thrombin (%)** |
| --- | --- | --- | --- | --- | --- | --- | --- | --- | --- |
| CAP1 | 2005 | f | 64 | 0 | 80 | 8.9 | 8.694 | 40,6 | 274 |
| CAP2 | 2004 | m | 78 | 0 | 75 | 10.4 | 6.83 | 32 | 202 |
| CAP3 | 2010 | m | 62 | 18 | 71 | 8.1 | 8.073 | 41 | 428 |
| CAP4 | 2011 | f | 62 | 12 | 86 | 13.8 | 7.6383 | 37 | 310 |
| CAP5 | 2005 | f | 40 | 0 | 96 | 6 | 8.7561 | 40,9 | 194 |
| CAP6 | 2007 | m | 71 | 0 | 64 | 9.1 | 8.4456 | 42 | 173 |
| CAP7 | 2008 | f | 60 | 0 | 117 | 26.11 | 6.89 | 34 | 411 |
| CAP8 | 2009 | m | 65 | 0 | 88 | 18.92 | 7.45 | 37 | 366 |
| CAP9 | 2012 | m | 76 | 15 | 99 | 19.8 | 8.4 | 39 | 384 |
| CAP10 | 2005 | f | 68 | 0 | 111 | np | np | np | np |
| CAP11 | 2011 | f | 73 | 13 | 94 | 7.1 | 7.7004 | 39 | 400 |
| CAP12 | 2005 | f | 69 | 0 | 103 | 13.4 | 8.5698 | 42 | 295 |
| CAP13 | 2012 | m | 78 | 20 | 80 | 14.8 | 8.4 | 41 | 213 |
| CAP14 | 2011 | f | 66 | 0 | 99 | 9.7 | 8.4456 | 41 | 421 |
| CAP15 | 2012 | m | 65 | 16 | 86 | 18.9 | 9.8 | 46 | 255 |
| CAP16 | 2009 | m | 35 | 0 | 117 | 11.3 | 9.315 | 44 | 76 |
| CAP17 | 2007 | m | 30 | 0 | 71 | 3.1 | 9.6255 | 47 | 160 |
| CAP18 | 2005 | m | 66 | 0 | 104 | 11.8 | 8.44 | 45 | 321 |
| CAP19 | 2009 | m | 69 | 0 | 85 | 9.7 | 9.19 | 46 | 286 |
| CAP20 | 2005 | f | 53 | 0 |  | 10 | 9.2529 | 43 | 556 |
| CAP21 | 2005 | f | 36 | 0 | 94 | 12.34 | 8.38 | 27 | 466 |
| CAP22 | 2011 | m | 71 | 15 | 80 | 12.5 | 7.5762 | 36 | 324 |
| CAP23 | 2010 | f | 71 | 12 | 90 | 15.5 | 8.1 | 39 | 302 |
| CAP24 | 2006 | f | 86 | 0 | 73 | 16.8 | 6.4584 | 32 | 438 |
| CAP25 | 2011 | f | 48 | 18 | 72 | 11.1 | 7.2657 | 36 | 492 |
| CAP26 | 2008 | m | 32 | 0 | 136 | 8.6 | 9.4392 | 43 | 230 |
| CAP27 | 2005 | f | 62 | 0 | 80 | 9 | 8.56 | 41 | 273 |
| CAP28 | 2005 | m | 63 | 0 | 119 | 4.51 | 8.01 | 37 | 109 |
| CAP29 | 2006 | m | 62 | 0 | 105 | 5.02 | 9.37 | 44 | 219 |
| CAP30 | 2004 | m | 29 | 0 | 105 | 10 | 10.8675 | 52 | 193 |
| **Statistics** | | | | | | | | | |
| female | 14 | | | | | | | | |
| male | 16 | | | | | | | | |
|  | minimum |  | 29 |  | 64 | 3 | 6 | 27 | 76 |
|  | Maximum |  | 86 |  | 136 | 26 | 11 | 52 | 556 |
|  | mean ± SD: |  | 60 ± 15.2 |  | 92 ± 17 | 12 ± 5 | 8 ± 0.1 | 40 ± 5 | 302 ± 116 |

np =not provided, SD = standard deviation

**Table S2:**  Differentially expressed proteins in a significantly lower or in a higher content when the PBMC were challenged with biofilms compared to the planktonic *S. aureus.*

| **Gene name** | **Protein name** | ***q*-value** | **Difference** |
| --- | --- | --- | --- |
| **PBMCs secretome challenged with planktonic *S. aureus*** | | | |
| HNRNPA2B1 | Heterogeneous nuclear ribonucleoproteins A2/B1 | 0.00122509618384282 | -8.10610771179199 |
| HK3 | Hexokinase-3 | 0.00138559797052849 | -4.55558904012045 |
| LSM3 | U6 snRNA-associated Sm-like protein LSm3 | 0.00225081976785979 | -3.68449211120605 |
| SND1 | Staphylococcal nuclease domain-containing protein 1 | 0.00232003026617817 | -3.72782198588054 |
| TPM3 |  | 0.00237272466422697 | -6.95476849873861 |
| OSTF1 | Osteoclast-stimulating factor 1 | 0.00241571821431637 | -4.55930773417155 |
| CUL3 | Cullin-3 | 0.00297950100523013 | -2.68819999694824 |
| LMNB2 | Lamin-B2 | 0.00320025507489868 | -4.59950574239095 |
| AIF1 | Allograft inflammatory factor 1 | 0.00324897549029276 | -3.69183858235677 |
| HNRNPC | Heterogeneous nuclear ribonucleoproteins C1/C2 | 0.00338688862703733 | -5.15377680460612 |
| PDCD6IP | Programmed cell death 6-interacting protein | 0.00383067671979529 | -4.72972106933594 |
| HCLS1 | Hematopoietic lineage cell-specific protein | 0.00383170580134592 | -4.53725306193034 |
| HSPA4 | Heat shock 70 kDa protein 4 | 0.00397016661393568 | -4.87321408589681 |
| NACA | Nascent polypeptide-associated complex subunit alpha, muscle-specific form | 0.00397016661393568 | -3.76478640238444 |
| MSN | Moesin | 0.00397016661393568 | -3.49235725402832 |
| HNRNPD | Heterogeneous nuclear ribonucleoprotein D0 | 0.00402990778790762 | -5.79779052734375 |
| GMFG | Glia maturation factor gamma | 0.0041987210073388 | -4.49427159627279 |
| SH3BP1 | SH3 domain-binding protein 1 | 0.00437467291374116 | -3.69127400716146 |
| ECHDC1 | Ethylmalonyl-CoA decarboxylase | 0.00437467291374116 | -2.885955174764 |
| BLVRB | Flavin reductase (NADPH) | 0.00474064582308963 | -3.57093556722005 |
| SLC9A3R1 | Na(+)/H(+) exchange regulatory cofactor NHE-RF1 | 0.00547121576384012 | -4.84702555338542 |
| CRK | Adapter molecule crk | 0.00547121576384012 | -2.83344841003418 |
| HNRNPA3 | Heterogeneous nuclear ribonucleoprotein A3 | 0.00581641911855921 | -2.21422894795736 |
| CAST | Calpastatin | 0.00665365209343952 | -3.59560648600261 |
| ACTL6A | Actin-like protein 6A | 0.00778497518249827 | -3.54909451802571 |
| PA2G4 | Proliferation-associated protein 2G4 | 0.00859712725982617 | -3.31376393636068 |
| Septin7 | Septin-7 | 0.00894782287908858 | -5.48598798116048 |
| HNRNPDL | Heterogeneous nuclear ribonucleoprotein D-like | 0.00895410925881499 | -3.21225992838542 |
| EEF1D | Elongation factor 1-delta | 0.0091010170650809 | -4.28445307413737 |
| Tenascin-X | Tenascin-X | 0.00929738162703745 | -4.20404688517252 |
| RANBP1 | Ran-specific GTPase-activating protein | 0.00948448416433658 | -3.33571434020996 |
| PRKAR1A | cAMP-dependent protein kinase type I-alpha regulatory subunit | 0.00977649771193105 | -3.46152877807617 |
| NAP1L4 | Nucleosome assembly protein 1-like 4 | 0.0100082590281251 | -4.51067733764648 |
| XPO1 | Exportin-1 | 0.0101581959738904 | -2.68554433186849 |
| ST13 | Putative protein FAM10A4 | 0.0103952265833467 | -4.22672780354818 |
| SRI | Sorcin | 0.0104994471138748 | -2.5242436726888 |
| SEC23A | Protein transport protein Sec23A | 0.01105117914789 | -3.47557067871094 |
| NCF1C | Putative neutrophil cytosol factor 1C | 0.0123006181719783 | -3.57029787699382 |
| HMHA1 | Minor histocompatibility protein HA-1 | 0.0132159145324689 | -3.00732040405273 |
| UCHL3 | Ubiquitin carboxyl-terminal hydrolase isozyme L3 | 0.0132948418570147 | -3.24047152201334 |
| SRSF1 | Serine/arginine-rich splicing factor 1 | 0.0134321693477346 | -2.22571627298991 |
| STK10 | Serine/threonine-protein kinase 10 | 0.0138816852171251 | -2.42886861165364 |
| STIP1 | Stress-induced-phosphoprotein 1 | 0.0149877971508931 | -2.20783869425456 |
| UBA7 | Ubiquitin-like modifier-activating enzyme 7 | 0.0158781353978281 | -3.3315060933431 |
| EIF2S2 | Eukaryotic translation initiation factor 2 subunit 2 | 0.0166947635412399 | -3.02618153889974 |
| COPS2 | COP9 signalosome complex subunit 2 | 0.0167952466259627 | -1.78138287862142 |
| APOBR | Apolipoprotein B receptor | 0.01680710686694 | -2.90913263956706 |
| PACSIN2 | Protein kinase C and casein kinase substrate in neurons protein 2 | 0.0170552071562728 | -3.32851537068685 |
| EEF1B2 | Elongation factor 1-beta | 0.0175703507409862 | -6.49446296691895 |
| SFPQ | Splicing factor, proline- and glutamine-rich | 0.0179598601503734 | -3.85586802164714 |
| PSME2 | Proteasome activator complex subunit 2 | 0.0198651481307435 | -3.89208348592123 |
| USP14 | Ubiquitin carboxyl-terminal hydrolase 14 | 0.0199065307847321 | -3.43786684672038 |
| PCNA | Proliferating cell nuclear antigen | 0.0208943512153932 | -2.94668579101563 |
| RPRD1B | Regulation of nuclear pre-mRNA domain-containing protein 1B | 0.0215541788345109 | -2.16319338480632 |
| PABPC1 | Polyadenylate-binding protein 1 | 0.0218196157134224 | -3.74851671854655 |
| ADD1 | Alpha-adducin | 0.0219294089277804 | -2.27154223124186 |
| AK2 | Adenylate kinase 2, mitochondrial | 0.0228134296314419 | -4.87518501281738 |
| PDAP1 | 28 kDa heat- and acid-stable phosphoprotein | 0.0228134296314419 | -2.93706448872884 |
| USP5 | Ubiquitin carboxyl-terminal hydrolase 5 | 0.0237584607025613 | -3.65934308369955 |
| DAK | Bifunctional ATP-dependent dihydroxyacetone kinase/FAD-AMP lyase (cyclizing) | 0.0250447963809251 | -4.49803733825684 |
| CFL1 | Cofilin-1 | 0.0251997897861103 | -3.29045041402181 |
| CNDP2 | Cytosolic non-specific dipeptidase | 0.0266458795699423 | -4.11043039957682 |
| CAPZA1 | F-actin-capping protein subunit alpha-1 | 0.026823108332479 | -3.28817176818848 |
| RNH1 | Ribonuclease inhibitor | 0.0268241437348965 | -1.26825777689616 |
| PSMD4 | 26S proteasome non-ATPase regulatory subunit 4 | 0.0271481128981753 | -1.82021649678548 |
| WAS | Wiskott-Aldrich syndrome protein | 0.0278833141371528 | -3.33872667948405 |
| PCBP1 | Poly(rC)-binding protein 1 | 0.0297264843622919 | -3.31076367696127 |
| MTPN | Myotrophin | 0.0299691658799691 | -5.6730162302653 |
| ENO1 | Alpha-enolase | 0.0320825341237851 | -1.18041928609212 |
| LZIC | Protein LZIC | 0.0324192030522067 | -4.23792394002279 |
| UFM1 | Ubiquitin-fold modifier 1 | 0.0324192030522067 | -3.21223704020182 |
| TXNL1 | Thioredoxin-like protein 1 | 0.0329158070126917 | -3.00758870442709 |
| PRDX5 | Peroxiredoxin-5 | 0.0329335109103681 | -5.48060862223307 |
| APBB1IP | Amyloid beta A4 precursor protein-binding family B member 1-interacting protein | 0.0329335109103681 | -1.75470288594564 |
| HNRNPK | Heterogeneous nuclear ribonucleoprotein K | 0.0331336808820948 | -2.84608713785807 |
| IL16 | Interleukin-16 | 0.0351401202336949 | -3.82675806681315 |
| PGK1 | Phosphoglycerate kinase 1 | 0.0364064560643602 | -3.61030197143555 |
| RBM8A | RNA-binding protein 8A | 0.0364064560643602 | -3.31323051452637 |
| IST1 | IST1 homolog | 0.0365839080826411 | -2.97500356038411 |
| ZYX | Zyxin | 0.0384886044804208 | -5.33604685465495 |
| SAFB2 | Scaffold attachment factor B2 | 0.03870437507837 | -2.86786460876465 |
| HSPB1 | Heat shock protein beta-1 | 0.0394799109341325 | -4.79301261901855 |
| PPA1 | Inorganic pyrophosphatase | 0.0417428300126576 | -2.61240450541178 |
| CCT8 | T-complex protein 1 subunit theta | 0.0422651203330631 | -2.83065223693848 |
| **PBMCs secretome challenged with *S. aureus* biofilm** | | | |
| NFS1 | Cysteine desulfurase | 0.00122509618384282 | 4.57199096679688 |
| DPP7 | Dipeptidyl peptidase 2 | 0.00122509618384282 | 3.44661649068196 |
| PRPF8 | Pre-mRNA-processing-splicing factor 8 | 0.00122509618384282 | 2.38769467671712 |
| NDUFV2 | NADH dehydrogenase [ubiquinone] flavoprotein 2 | 0.00122509618384282 | 1.47683525085449 |
| GIMAP7 | GTPase IMAP family member 7 | 0.00125629237862989 | 1.59690539042155 |
| TES | Testin | 0.00173345808679002 | 3.74503135681152 |
| GGCT | Gamma-glutamylcyclotransferase | 0.00179258164364405 | 4.65143330891927 |
| RWDD1 | RWD domain-containing protein 1 | 0.00222940224916641 | 2.76996676127116 |
| HSPA9 | Stress-70 protein, mitochondrial | 0.00268723171806932 | 8.80506833394368 |
| RPS8 | 40S ribosomal protein S8 | 0.00268723171806932 | 2.73138745625814 |
| ACTG1 |  | 0.00270854196253852 | 2.88363965352377 |
| MAN2B1 | Lysosomal alpha-mannosidase;Lysosomal alpha-mannosidase A,B,C,D,E peptides | 0.00355508734640218 | 3.00924301147461 |
| DLST | Dihydrolipoyllysine-residue succinyltransferase component of 2-oxoglutarate dehydrogenase complex | 0.00402990778790762 | 3.78886985778809 |
| DLAT | Acetyltransferase component of pyruvate dehydrogenase complex | 0.00402990778790762 | 3.04300753275553 |
| NIPSNAP3A | Protein NipSnap homolog 3A | 0.00419524192517497 | 4.33978716532389 |
| EFHD2 | EF-hand domain-containing protein D2 | 0.00706683290479033 | 0.947721481323242 |
| RPS2 | 40S ribosomal protein S2 | 0.00936505003372187 | 2.43293698628743 |
| TRPV3 | Transient receptor potential cation channel subfamily V member 3 | 0.00939019774921722 | 4.19886589050293 |
| HSP90AB1 | Heat shock protein HSP 90-beta | 0.00941346035489018 | 1.10247993469238 |
| FAHD1 | Acylpyruvase FAHD1 | 0.0125047032092976 | 2.57362492879232 |
| TSNAX | Translin-associated protein X | 0.0135848123766292 | 1.96796417236328 |
| AP3B1 | AP-3 complex subunit beta-1 | 0.0139331305578253 | 2.88084093729655 |
| HIST2H3PS2 | Histone H3 | 0.0154727508092478 | 4.84566307067871 |
| MDH2 | Malate dehydrogenase, mitochondrial | 0.0165837151503223 | 1.65378252665202 |
| HSP90AA1 | Heat shock protein HSP 90-alpha | 0.0175320984445254 | 1.18663597106934 |
| EIF5B | Eukaryotic translation initiation factor 5B | 0.0182399369205337 | 1.70312436421712 |
| RPL22 | 60S ribosomal protein L22 | 0.0190231460563828 | 2.80422655741374 |
| PRKDC | DNA-dependent protein kinase catalytic subunit | 0.0237584607025613 | 3.03001085917155 |
| RPS3 | 40S ribosomal protein S3 | 0.025850393318349 | 3.23059844970703 |
| Septin11 | Septin-11 | 0.0268241437348965 | 2.41893132527669 |
| U2AF2 | Splicing factor U2AF 65 kDa subunit | 0.0271481128981753 | 1.97892634073893 |
| DPYSL2 | Dihydropyrimidinase-related protein 2 | 0.0351401202336949 | 0.886943817138672 |
| SERPINA3 | Alpha-1-antichymotrypsin | 0.0365839080826411 | 1.44390869140625 |
